# Simulated Conversations With Virtual Humans to Improve Patient-Provider Communication and Reduce Unnecessary Prescriptions for Antibiotics: A Repeated Measure Pilot Study

**DOI:** 10.2196/mededu.6305

**Published:** 2017-04-19

**Authors:** Antoinette Schoenthaler, Glenn Albright, Judith Hibbard, Ron Goldman

**Affiliations:** ^1^ Center for Healthful Behavior Change Department of Population Health NYU School of Medicine New York, NY United States; ^2^ Baruch College City University of New York New York, NY United States; ^3^ Kognito New York, NY United States; ^4^ Health Policy Research Group University of Oregon Eugene, OR United States

**Keywords:** simulation training, health communication, patient activation, motivational interviewing, decision making

## Abstract

**Background:**

Despite clear evidence that antibiotics do not cure viral infections, the problem of unnecessary prescribing of antibiotics in ambulatory care persists, and in some cases, prescribing patterns have increased. The overuse of antibiotics for treating viral infections has created numerous economic and clinical consequences including increased medical costs due to unnecessary hospitalizations, antibiotic resistance, disruption of gut bacteria, and obesity. Recent research has underscored the importance of collaborative patient-provider communication as a means to reduce the high rates of unnecessary prescriptions for antibiotics. However, most patients and providers do not feel prepared to engage in such challenging conversations.

**Objectives:**

The aim of this pilot study was to assess the ability of a brief 15-min simulated role-play conversation with virtual humans to serve as a preliminary step to help health care providers and patients practice, and learn how to engage in effective conversations about antibiotics overuse.

**Methods:**

A total of 69 participants (35 providers and 34 patients) completed the simulation once in one sitting. A pre-post repeated measures design was used to assess changes in patients’ and providers’ self-reported communication behaviors, activation, and preparedness, intention, and confidence to effectively communicate in the patient-provider encounter. Changes in patients’ knowledge and beliefs regarding antibiotic use were also evaluated.

**Results:**

Patients experienced a short-term positive improvement in beliefs about appropriate antibiotic use for infection (*F*_1,30_=14.10, *P*=.001). Knowledge scores regarding the correct uses of antibiotics improved immediately postsimulation, but decreased at the 1-month follow-up (*F*_1,30_=31.16, *P*<.001). There was no change in patient activation and shared decision-making (SDM) scores in the total sample of patients (*P*>.10) Patients with lower levels of activation exhibited positive, short-term benefits in increased intent and confidence to discuss their needs and ask questions in the clinic visit, positive attitudes regarding participation in SDM with their provider, and accurate beliefs about the use of antibiotics (*P*<.10). The results also suggest small immediate gains in providers’ attitudes about SDM (mean change 0.20; *F*_1,33_*=* 8.03, *P*=.01).

**Conclusions:**

This pilot study provided preliminary evidence on the efficacy of the use of simulated conversations with virtual humans as a tool to improve patient-provider communication (ie, through increasing patient confidence to actively participate in the visit and physician attitudes about SDM) for engaging in conversations about antibiotic use. Future research should explore if repeated opportunities to use the 15-min simulation as well as providing users with several different conversations to practice with would result in sustained improvements in antibiotics beliefs and knowledge and communication behaviors over time. The results of this pilot study offered several opportunities to improve on the simulation in order to bolster communication skills and knowledge retention.

## Introduction

The economic and clinical consequences of antibiotic overuse are numerous and can lead to increased medical costs due to unnecessary hospitalizations [1], antibiotic resistance [2], disruption of gut bacteria [3], and more recently obesity [4]. Despite many individual and organizational efforts to address the unnecessary prescribing of antibiotics in ambulatory care, the problem persists, and in some cases, prescribing patterns have increased [5,6]. In fact, recent evidence shows that up to 30% of antibiotics were inappropriately prescribed in 2010-2011, with a majority of these prescriptions being given for sinusitis [7].

Patients and health care providers often express frustration engaging in conversations about challenging or sensitive topics such as the overuse of antibiotics for treating viral infections within the clinic encounter. A review of the evidence shows that most antibiotics for viral infections are not prescribed as the result of clinical evidence but rather given in response to patient demands or lack of training in the appropriate guidelines among health care providers [8,9]. However, findings from intervention studies suggest that the provision of treatment guidelines to physicians and patient education alone are insufficient for reducing unnecessary prescribing of antibiotics [10,11]. Rather, recent research has underscored the importance of incorporating collaborative, patient-centered communication, and SDM into the medical visit as a means to reduce the high rates of unnecessary prescriptions for antibiotics [12]. In 2012, the Choosing Wisely campaign was launched by the America Board of Internal Medicine (ABIM) Foundation, along with Consumer Reports and the Robert Wood Johnson Foundation (RWJF) in an effort to facilitate collaborative patient-provider communication aimed at reducing treatment overuse and waste [13]. Patient engagement, in particular, was regarded as one of the primary methods for reducing antibiotic overuse in the context of the Choosing Wisely campaign. The February 2013 issue of *Health Affairs* was entirely devoted to patient engagement practices and cited strong evidence that “patients who are more actively involved in their health care experience have better health outcomes and incur lower costs” [14]. Similarly, Greene and Hibbard [15] found that interventions that address individual levels of activation and build skills and confidence are effective in activating patients, thereby reducing cost and improving care quality and outcomes. Finally, sound communication in health care interventions and informatics has been shown to allow providers and patients to make sense of the information provided and make sustained changes [16,17].

Building on this evidence, this pilot study examined whether a 10-15 min simulated role-play conversation with a virtual human, one for providers and one for patients, can facilitate the development of collaborative communication skills, knowledge, and confidence of patients and providers to effectively manage conversations regarding overprescribing of antibiotics for viral infections. Specifically, we hypothesized that use of the simulation would result in improvements in (1) patients’ knowledge and attitudes toward antibiotic usage; (2) patient activation; (3) patients’ and providers’ attitudes toward and preference for SDM; (4) providers’ perception of patient engagement in their self-management; and (5) patients’ and providers’ confidence, preparedness, and behavioral intention to engage in conversations about antibiotics.

## Methods

### Participants

This pilot study used a single group repeated measures design. Patients were recruited from the Bellevue Ambulatory Care practice, a New York City-based public hospital-based primary care practice that serves predominately low-income minority patient populations. Patients were recruited through their previous participation in completed trials with one of the study authors at Bellevue Hospital. Patients were sent letters inviting them to participate in the study and a telephone number to call for more information. Study staff also called patients inviting them to participate. Patients were excluded from the study if they (1) were unable to give informed consent, (2) refused to participate, (3) were unable to speak and read in English, or (4) age <18 years. Primary care providers were affiliated with NYU Langone Medical Center, providing care across four health care facilities: Bellevue Hospital, Gouverneur Health, Veterans Affairs NY Harbor Health Care System’s New York Campus, and the NYU Faculty Group Practice. An email was sent to providers inviting them to participate in the study. All patients and providers provided written informed consent approved by the Institutional Review Board of New York University Langone Medical Center.

### Description of the Patient-Provider Communication Simulation

The 15-min simulation was developed by Kognito in collaboration with a group of experts in motivational interviewing, patient engagement, medical education, and antibiotics. In addition, over 25 patients and providers provided feedback during the development phase before the beta version was piloted in this study.

**Figure 1:**
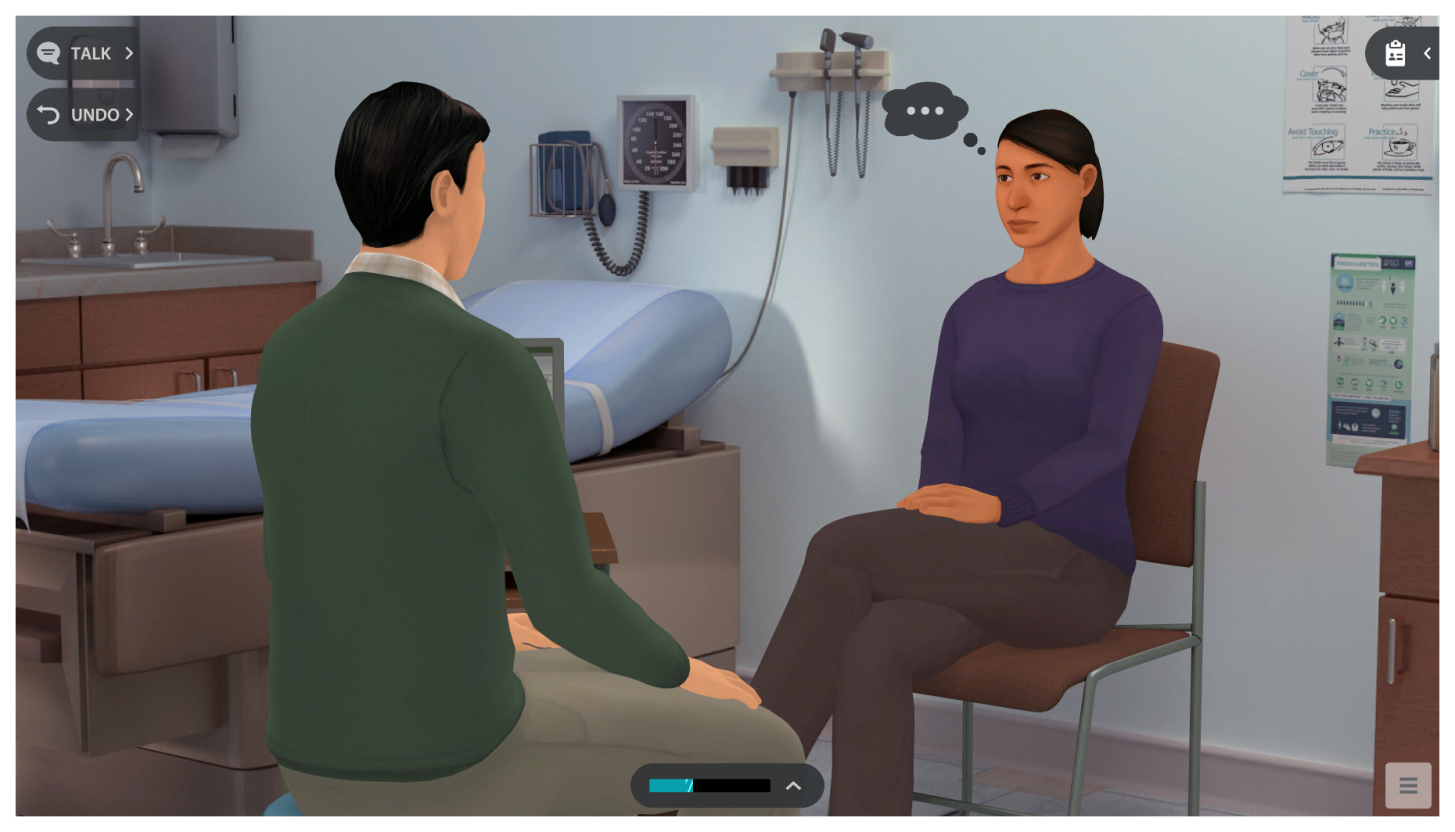
Dr Wei talking with his patient Laura.

For this study, both patients and health care providers engaged in a simulated conversation aimed at the overarching goal of improving collaborative patient-physician communication and SDM for antibiotic use. When patients accessed the simulation, they assume the role of Laura (the virtual patient) and engage in a conversation with Dr Wei (the virtual provider). Health care providers enter the simulation taking on the role of Dr Wei, who has to manage the conversation with the patient, Laura. At the beginning of the learning experience, participants view a brief movie that explains the backstory and their goals in the conversation. For example, participating providers are told that they will play the role of Dr Wei and conduct an office visit with Laura, a mom who has been coughing for a week and believes that antibiotics can help her get better quickly. Their goals in the conversation are to engage Laura in a conversation about her condition and health goals, and then to collaborate with her on a treatment plan that she understands and is motivated to follow all while expressing empathy, using plain language, checking understanding, and managing her repeating requests for antibiotics. Study patients who choose to play the role of the virtual patient are told that they will act as Laura in the conversation and decide what to say to the virtual physician, Dr Wei. Their goals in the conversation are to provide Dr Wei with a clear understanding of Laura’s illness, ask Dr Wei questions so that they understand everything he says, learn about the proper use of antibiotics, and to work with Dr Wei on a plan they both are satisfied with (Figure 1).

**Figure 2:**
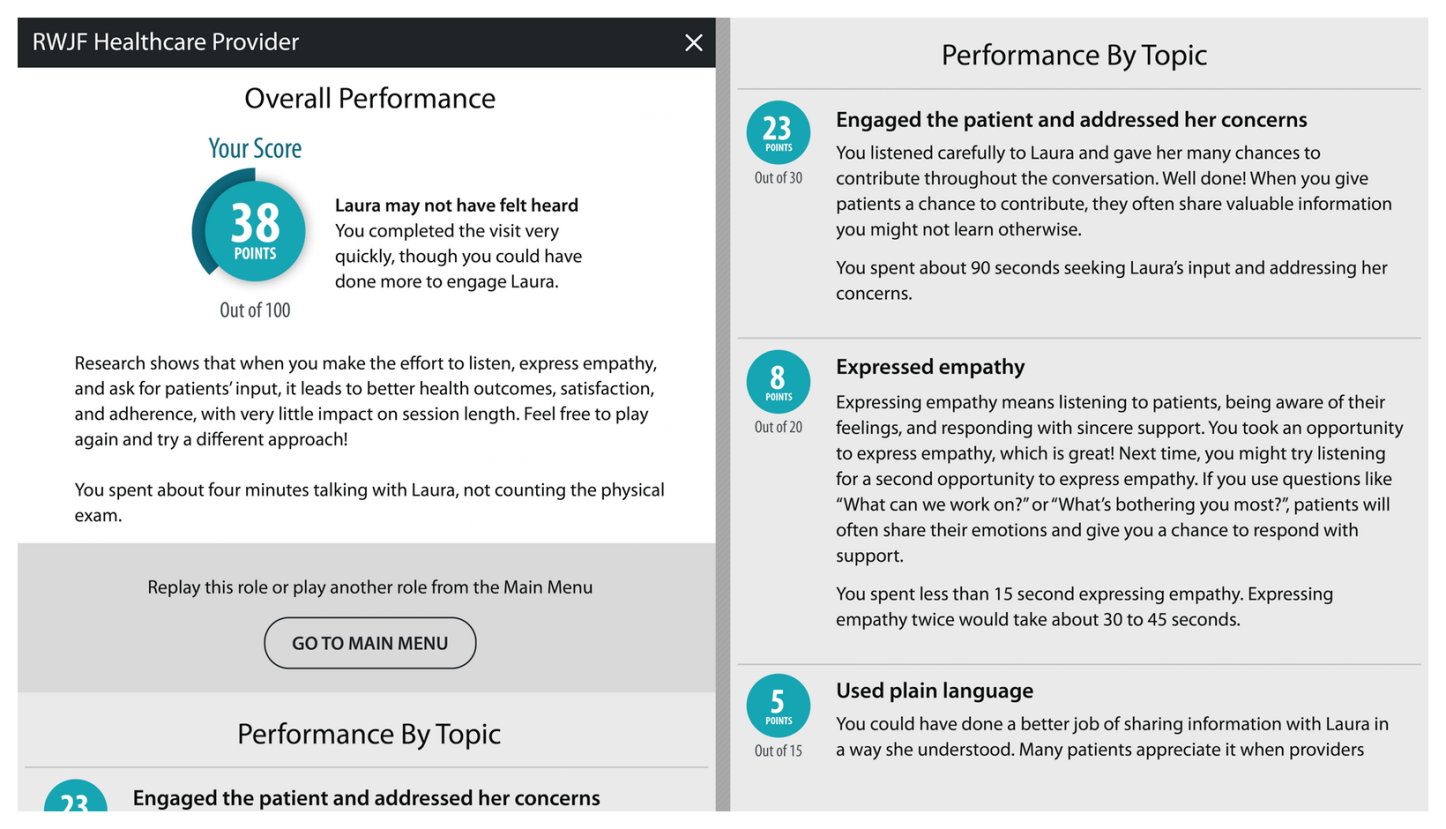
Performance dashboard provided to users who played the role of the provider. RWJF: Robert Wood Johnson Foundation.

At the end of the 15-min simulation, users view a brief movie where the virtual coach provides them with feedback on the decisions they made in the conversation. Then, they are provided with a performance dashboard that includes more detailed feedback on their performance. The information in the dashboard is based on the exact dialog decisions made by the learner during the conversation (Figure 2).

The simulation was designed using the Kognito Conversation Platform, an innovative group of development, delivery, application programming interface (API), data collection, and analytic technologies that integrates a behavior change model that employs the principles of neuroscience, social cognition, adult learning, applied game mechanics, and storytelling [18]. This learning model includes two parts: the first part is an instructional design component based on extensive research showing that skills are best learned when knowledge is actively constructed [19-23]. Learners are afforded multiple opportunities to actively make conversation decisions in a virtual environment where emphasis is placed on reducing extraneous cognitive load to create deeper and more meaningful unique pathways of experience on an individual level. The second part, the conversation component, involves integration of evidence-based communication strategies including motivational interviewing [24], mentalizing [25-27], empathy [28,29], empathic accuracy [30] (knowing what someone is feeling without feeling it yourself), and reappraisal strategy [31,32] (changing the way you interpret a situation). The conversation component is further augmented by aspects of social cognitive theory [33] for participants who are observing how the virtual characters interact and the consequences of those interactions in a contextually realistic environment, which can guide them in making real-life decisions.

In each simulation, a learner enters a risk-free practice environment, assumes a role (ie, health care provider, patient), and engages in a conversation with intelligent, fully animated, and emotionally responsive virtual characters that model human behavior. Virtual humans are coded to possess an individual personality and memory, and adapt their behaviors to the decisions of the learner throughout the conversation. Learners communicate with the virtual human by selecting from a dynamic menu of dialog options. Each option represents a specific conversation tactic based on communications skills that may be more or less effective or ineffective in accomplishing the learner’s goal (see Figure 1). The simulation, including dialog options, was developed with input from nationally recognized subject matter experts and end users as part of a comprehensive iterative process involving every aspect of simulation development, ranging from accuracy of content, integration of motivational interviewing strategies and other communication tactics, engaging and realistic storylines, virtual character development, and verbal and nonverbal responses. Once the learner chooses a dialog option, they see their avatar “perform” the dialog, and then observe the verbal and nonverbal response of the virtual human. A new set of dialog options then appears based on the specific tactic selected. If learners select choices that include being critical, judgmental, or labeling, for example, they will lose some of their interlocutor’s trust and willingness to talk openly. In these cases, a virtual coach provides personalized feedback and gives learners an opportunity to revise their choice. The virtual coach also provides feedback at the conclusion of the simulation based on the learner’s performance as they relate to the study objectives.

The relationship between dialog decisions made by the learner and the response of the emotionally responsive virtual human is controlled by a set of mathematical behavioral models and algorithms specifically designed to simulate real interactions with “types” of people presenting particular personality traits or conditions (ie, cold or cough). The algorithms ensure that learners are repeatedly exposed to target conversations and behavioral patterns as a way to develop skills.

### Study Measures

Self-report measures were administered at the baseline (presimulation), immediate postsimulation, and 1-month follow-up to participating patients and providers. The measures were designed to assess key aspects of patient-provider communication targeted in the simulation (ie, SDM), patient activation, patient antibiotic knowledge and beliefs, behavioral intentions, preparedness, and confidence to engage in challenging conversations, and satisfaction with the simulated encounter. In addition, exit interviews were conducted postsimulation to determine acceptability of the approach.

*Antibiotics beliefs and knowledge* was assessed for patients using a measure developed for medical students [34]. The knowledge items assessed the extent to which patients accurately understood the correct uses of antibiotics and the costs of using antibiotics (ie, antibiotics are effective at treating bacterial infections; Cronbach alpha=.59). Perceived belief items asked how much patients agreed or disagreed with commonly held attitudes regarding the use of antibiotics for infection (ie, I expect antibiotics when experiencing cold symptoms; I keep extra antibiotics at home for an emergency; *lower* scores indicate more accurate beliefs; Cronbach alpha=.75). Both scales were measured with a 4-point Likert scale (range: strongly disagree to strongly agree).

*Patient activation* was measured for patients with the *Patient Activation Measure (PAM)* developed by Hibbard et al [35,36]. The PAM assesses an individual’s knowledge, skill, and confidence for managing one’s own health and health care (Cronbach alpha=.89). In addition, the Clinician Support for Patient Activation Measure (CS-PAM) was used to assess clinician beliefs about the importance of patient participation in care (Cronbach alpha=.84) [37]. Both measures, each with 13 items were created using Rasch Analysis and produce a 0-100 score. The surveys were administered to participants (patients and providers, respectively) during the first in-person session (pre- and postsimulation) as well as during the 1-month follow-up session.

*SDM* was measured for patients and providers with the shared subscale from the *Patient-Provider Orientation Scale (PPOS)* [38,39]. This measure assessed the patient’s and provider’s attitudes about one another’s role in the encounter as it applies to the decision-making process (ie, patients should be treated as if they were partners with the doctor, equal in power and status). Responses were given on a 4-point Likert scale (range: strongly disagree to strongly agree, with *lower* scores indicating a higher degree of SDM; Cronbach alpha=.78). Providers also completed the *SDM-Q-doc* [40,41], which assessed the extent to which providers perceive that they engage patients in the treatment decision-making process. Responses were given on a 4-point Likert scale (range: completely disagree to completely agree; Cronbach alpha=.85). These surveys were administered to participants (patients and providers) during the in-person session (pre- and postsimulation) and during the 1-month follow-up session.

*Patient preferences for decision-making* were assessed with the decision-making subscale from the *Medical Communication Competence Scale (MCCS)* using a 4-point Likert scale (range: strongly disagree to strongly agree; Cronbach alpha=.71) [42]. The subscale assessed the extent to which patients preferred medical visits to be doctor-centric (ie, important medical decisions about your health should be made by the doctor, not you) versus patient-centered. The survey was administered to patients during the in-person session (pre- and postsimulation) and during the 1-month follow-up session.

*Confidence, preparedness, and behavioral intention to engage in challenging conversations* was a measure developed for this project with the intention to assess the preliminary effect of using the simulation on patients’ and providers’ behaviors and attitudes in the patient-provider clinic visit (Cronbach alpha=.97). All three attitudinal constructs were drawn from psychological frameworks aimed at understanding goal driven behavior and predicting future outcomes. This includes Reasoned Action Theory [43], which posits that behavioral beliefs and subjective norms are the antecedents to behavioral intention which is the direct precedent to behavior, and Bandura’s [44] integrative framework of personal efficacy or perceived behavior control where self-efficacy is both a direct and indirect predictor of behavior.

*For patients*, the preparedness items asked patients how prepared they felt to ask their doctor questions, express their concerns, and discuss treatment options as a result of using the simulation. The behavioral intent items asked patients to rate how likely they would ask questions in the clinic visit and give their doctor details about how their health is affecting them personally. The confidence items assessed patients’ level of confidence to ask questions, express their concerns even when the doctor does not ask, and engage in discussions about different treatment options. *For providers*, the preparedness items asked providers to rate the extent to which they felt *other doctors* would be better prepared to seek patients’ input, respond to patient emotion, have effective conversations about antibiotics, share information in a way patients understand, and invite patients to ask questions and participate in the conversation, as a result of the simulation. Behavioral intent items asked how likely other doctors would ask patients how their problems affects their everyday life and goals, engage in conversations about antibiotics, and invite patients to ask questions. Finally, the confidence items asked if doctors would be more confident to engage patients in conversations about their goals, respond to emotion, and have effective conversations about antibiotics. These questions were asked on a 4-point Likert scale (range: strongly disagree to strongly agree) immediately following the simulation and at the 1-month follow-up. In addition, providers were asked if the simulation influenced the way they work with patients in general and when speaking about antibiotics since completing the simulation 1 month ago.

*Participant sociodemographics* were measured at the baseline visit. Data on patients’ age, gender, education level, insurance status, and health literacy level [45] were collected. Provider data included age, gender, occupation (ie, internist, family medicine provider, nurse practitioner), rank (ie, attending, resident), degree, mean years at clinic, and exposure to previous communication skills training.

### Analysis

Descriptive statistics were generated for baseline patient and provider characteristics. Generalized Linear Models (GLM) using repeated measures analysis were used to analyze the pre-, post- and 1-month follow-up survey measures. Analyses were first run for the total sample and then repeated for the subset of participants, who were in the lower PAM levels at the baseline visit (presimulation) (PAM level 1 or 2; n=13). Independent *t* test was used to compare the study measures by provider rank (attending vs resident) at a single time point; GLM was used to compare scores across time using a time X rank interaction. Since this pilot study is exploratory, we used a *P* value <.1 to denote significant findings.

## Results

### Participant Characteristics

We recruited a total of 69 participants (35 providers and 34 patients); with a retention rate of 99% (68/69) (one patient was lost at follow-up). As shown in Table 1, approximately half of the patient sample was female (53%, 18/34), one-third had Medicaid (29%, 10/34), and the mean age was 57.6 years (standard deviation, SD 14.6). Two-thirds of patients reported having some college education or above (70%, 23/33) and a health literacy level equivalent to a high school degree (68%, 23/34). Approximately half of the provider sample was male (53%, 19/35), two-thirds were internists (63%, 22/35), and most had an MD (97%, 34/35), with a mean age of 40.34 years (SD 9.44). The average practice duration was 7.48 years (SD 6.53) at the clinic. Three-quarters of providers were attendings and the remaining one-quarter were residents. More than half (58%, 11/19) of the providers reported *some to quite a bit* previous exposure to training in communication skills (Table 1).

### Patient Results

#### The Effect of the Simulation on Patients’ Knowledge and Beliefs Regarding Antibiotic Use

At the presurvey assessment, the mean score on the beliefs subscale was 1.85 (range: 1-4; lower scores indicate more accurate beliefs). Immediately after completing the simulation, patients were significantly more likely to possess accurate beliefs about antibiotic use (mean change −0.11, *P*=.001). The improvement in accurate beliefs was maintained at the 1-month follow-up (mean 1.76; *F*_1,30_=14.10, *P*<.001). The mean score on the knowledge subscale at the presurvey assessment was 3.01 (range: 1-4; higher scores indicate more accurate knowledge). Knowledge about antibiotics significantly improved postsimulation (mean 3.26, *P*<.001). However, knowledge scores significantly decreased from the post assessment to the 1-month follow-up (mean 3.08; *F*_1,30_=31.16, *P*<.001).

#### The Effect of the Simulation on Patient Activation

The mean PAM score for the total patient sample was 63.60 (SD 15.39) at the presurvey assessment, 62.61 (SD 13.35) at the immediate postsurvey assessment, and 62.83 (SD 14.57) at the 1-month follow-up. Results of the repeated measures analysis showed no significant differences in PAM score across time (*F*_1,31_=1.86, *P*=.18; Table 2).

#### The Effect of the Simulation on Patients’ Perceptions of Shared Decision-Making

At the previsit assessment, patients not only tended to prefer their doctor make the final decisions about their care (mean 2.66, range 1-4; Table 2), but also believed that decision-making should be a collaborative process (mean 2.06, range 1-4; note: lower scores indicate a more collaborative encounter). Although not significant, patients reported an increased preference for being actively involved in the decision-making process with their doctor after completing the simulation and at the 1-month follow-up (mean change 0.19 and 0.17, respectively; *F*_1,31_=1.94, *P*=.17). Attitudes about patients’ role in decision-making did not change across time (*F*_1,28_=1.86, *P*=.18).

#### The Effect of the Simulation on Patients’ Level of Preparedness, Confidence, and Behavioral Intent to Actively Participate in a Clinic Encounter

After completing the simulation, patients reported feeling prepared to actively participate in a future medical visit with their provider (mean 3.35, range 1-4), which did not significantly change at the 1-month follow-up (*P*=.47). In addition, after completing the simulation, patients’ agreed that they were more likely to ask questions in the clinic visit and give their doctor details about how their health is affecting them personally (mean 3.24, range 1-4), which was maintained at the 1-month follow-up (mean 3.31; *P*=.43). Finally, at both the post survey and 1-month follow-up, patients agreed that they felt more confident in their ability to ask questions, tell their provider how they are feeling, and work with their provider to make a treatment plan. There was no difference in scores across the time points (*P*=.12; Table 2).

**Table 1:** Participant characteristics.

Characteristics			Mean (SD^a^) or n (%)
Patient characteristics			
	Age		57.62 (14.57)
	**Gender**		
		Male	16 (47)
	**Literacy levels**		
		7th to 8th grade	11 (32)
		High school	23 (68)
	**Education level**		
		Less than high school	1 (3)
		high school/GED^b^/technical	9 (27)
		Some college	8 (24)
		College degree	15 (46)
	**Patient insurance**		
		Private	9 (27)
		Medicare	10 (29)
		Medicaid	10 (29)
		None	2 (6)
		Other	3 (9)
Provider characteristics			
	Age		40.34 (9.44)
	**Gender**			
		Male	19 (54)
	**Occupation**		
		Physician/family medicine	12 (34)
		Physician/internal medicine	22 (63)
		Nurse practitioner	1 (3)
	**Rank**		
		Attending	26 (74)
		Resident	9 (26)
	**Degree**		
		MD^c^/DO^d^	34 (97)
		NP^e^	1 (3)
		Mean years at clinic	7.48 (6.53)
	**Provider communication skills training**		
		A little	8 (42)
		Some	7 (37)
		Quite a bit	4 (21)

^a^SD: standard deviation.

^b^GED: General Educational Development.

^c^MD: Doctor of Medicine.

^d^DO: Doctor of Osteopathy.

^e^NP: nurse practitioner.

**Table 2:** Comparisons of patient survey responses across all three time points.

Patient measures	Response range	Presimulation mean (SD^a^)	Postsimulation mean (SD)	1-month follow-up mean (SD)	*F*	*P*
PAM^b^ score	0-100	63.60 (15.39)	62.61 (13.35)	62.83 (14.57)	1.86	.18
Antibiotic beliefs	1-4^c^	1.85 (0.42)	1.74 (0.41)	1.76 (0.48)	14.10	.001
Antibiotic knowledge	1-4	3.01 (0.42)	3.26 (0.43)	3.08 (0.46)	31.16	<.001
Decision-making preference	1-4^c^	2.66 (0.73)	2.85 (0.73)	2.83 (0.78)	1.94	.17
Patient-provider orientation: shared power	1-4^c^	2.06 (0.44)	2.01 (0.40)	2.02 (0.41)	1.86	.18
Preparedness to act	1-4	-	3.35 (0.59)	3.25 (0.53)	0.74	.47
Behavioral intent	1-4	-	3.24 (0.57)	3.31 (0.52)	0.81	.43
Confidence to act	1-4	-	3.32 (0.61)	3.34 (0.57)	−1.62	.12

^a^SD: standard deviation.

^b^PAM: Patient Activation Measure.

^c^Lower scores indicate more accurate beliefs about antibiotics and shared power in the clinic encounter.

#### Subanalysis of Patients With Low PAM Scores

Results of the subanalysis showed that patients with low PAM scores demonstrated similar improvements in accurate beliefs regarding antibiotic use at the postsurvey (mean 2.04) and 1-month follow-up (mean 2.01; *F*_1,9_=10.88, *P*=.01). Moreover, knowledge about antibiotics significantly improved postsimulation (mean 3.23) and then decreased at the 1-month follow-up (mean 2.89; *F*=28.53_1,10_, *P*<.001). At the baseline (presimulation) assessment, patients in the lower PAM levels tended to prefer their doctor make the final decisions about their care at the previsit assessment (mean 2.58) as well as somewhat agree that patients and providers should be equal partners in the decision-making process (mean 2.40). After completing the simulation and at the 1-month follow-up, patients reported a significant improvement in attitudes about their role in decision-making (*F*_1,9_=17.19, *P*=.002). Although patients’ preference for being actively involved in the decision-making process with their provider improved across time (mean 2.69 [postsimulation] and 2.64 [1-month]), the change was not significant (*F*_1,10_=0.45, *P*=.52). Similar to the total sample, patients in the low PAM levels did not exhibit changes in PAM scores across time (*P*=.58; Table 3).

Patients with low PAM scores also agreed that they felt better prepared to ask their doctor questions, express their concerns, and discuss treatment options after completing the simulation (mean 3.24) and at the 1-month follow-up (mean 3.33). There was no significant differences in the mean scores across time (*F*_1,6_=−0.55, *P*=.60; Table 3). Similarly, these patients agreed that they were more likely to ask questions in the clinic visit and give their doctor details about how their health is affecting them personally (mean 3.25; range 1-4), which was increased significantly at the 1-month follow-up (mean 3.50; *t*_5_=−2.24, *P*=.08). Finally, in terms of confidence to engage in the conversation, patients agreed that they felt more confident in their ability to ask questions at the post survey (mean 2.93), which significantly increased at the 1-month follow-up (mean 3.33; *t*_5_=−2.34, *P*=.07; Table 3).

**Table 3:** Comparisons of low PAM patient survey responses across all three time points (n=13).

Patient measures	Response range	Presimulation mean (SD^a^)	Postsimulation mean (SD)	1-month follow-up mean (SD)	*F*	*P*
PAM^b^ score	0-100	39.00 (5.66)	40.90 (6.79)	40.90 (5.72)	0.32	.58
Antibiotic beliefs	1-4^c^	2.40 (0.20)	2.04 (0.27)	2.01 (0.44)	10.88	.01
Antibiotic knowledge	1-4	2.86 (0.25)	3.23 (0.39)	2.89 (0.23)	28.53	<.001
Decision-making preference	1-4	2.58 (0.49)	2.69 (0.48)	2.64 (0.71)	0.45	.52
Patient-provider orientation: shared power	1-4^c^	2.40 (0.20)	2.27 (0.16)	2.17 (0.30)	17.19	.002
Preparedness to act	1-4	-	3.24 (0.71)	3.33 (0.27)	−0.55	.60
Behavioral intent	1-4	-	3.25 (0.42)	3.50 (0.32)	−2.24	.08
Confidence to act	1-4	-	2.93 (0.52)	3.33 (0.50)	−2.34	.07

^a^SD: standard deviation.

^b^PAM: Patient Activation Measure.

^c^Lower scores indicate more accurate beliefs and shared power in the clinic encounter.

### Provider Results

#### The Effect of the Simulation of Providers’ Perception of Patient Engagement in Their Self-Management

Before engaging with the simulation, providers held high positive beliefs about patient’s involvement in their self-management (mean 78.19, range 0-100). These ratings remained high (mean 76.47) immediately after completing the simulation as well as at the 1-month follow-up (mean 77.15). There were no significant differences across time (*F*_1,34_=0.11, *P*=.74; Table 4).

#### The Effect of the Simulation on Providers’ Level of Preparedness, Confidence, and Behavioral Intent to Effectively Communicate With Patients

Immediately after completing the simulation, participating providers felt that doctors would be better prepared to have an effective conversation with patients (mean 3.45), actively engage patients in the conversation (mean 3.48), and feel confident in their abilities to engage and respond to patients’ biomedical and psychosocial concerns (mean 3.33). Similar to the postsimulation results, providers continued to agree that doctors would be better prepared, confident, and able to effectively engage in conversations about antibiotics, respond to patient emotion, and invite patients to be active participants in the medical encounter. All providers also felt doctors would be more prepared to have an effective conversation about antibiotics with patients (Table 4). There was no differences in scores across time for each of the measures (*P*>.10).

#### The Effect of the Simulation on Providers’ Perceptions of Shared Decision-Making

Before completing the simulation, providers felt that they already engaged patients in the shared decision-making process (mean 3.24, range 1-4) and that decision-making process should be a collaborative process (mean 1.82, range 1-4; lower scores indicate more collaboration). Immediately after completing the simulation, there was a significant increase in providers’ attitudes about patients’ collaborative involvement in the shared decision-making process (mean 1.62, *F*_1,33_=8.03, *P*=.01; Table 4). However, this score returned to the baseline level at the 1-month follow-up (mean 1.82). There was no change in providers’ perceived use of shared decision-making skills with patients from the presurvey to 1-month follow-up (*F*_1,34_=1.61, *P*=.21).

**Table 4:** Comparisons of provider survey responses across all three time points.

Provider measures	Response range	Presimulation mean (SD^a^)	Postsimulation mean (SD)	1-month follow-up mean (SD)	*F*	*P*
CS-PAM^b^	1-100	78.19 (13.02)	76.47 (13.34)	77.15 (14.44)	0.11	.74
Shared decision-making	1-4	3.24 (0.38)	-	3.35 (0.38)	1.61	.21
Patient-provider orientation: shared power	1-4^c^	1.82 (0.37)	1.62 (0.32)	1.82 (0.39)	8.03	.01
Satisfaction	1-4	-	3.25 (0.28)	-	-	-
Preparedness to act	1-4	-	3.45 (0.50)	3.34 (0.44)	−0.36	.73
Behavioral intent	1-4	-	3.48 (0.47)	3.42 (0.44)	−0.84	.42
Confidence to act	1-4	-	3.33 (0.60)	3.31 (0.41)	−1.30	.22

^a^SD: standard deviation.

^b^CS-PAM: Clinician Support for Patient Activation Measure.

^c^Lower scores indicate shared power in the clinic encounter.

#### Findings by Provider Rank

In cross-sectional analysis, comparing the data by provider rank (resident vs attending), there were no significant differences between the groups before completing the simulation. At the 1-month follow-up, attendings were more likely to agree that patients should be actively involved in the shared decision-making process (mean 3.38 vs 3.28, *P*=.01), whereas residents were more likely to believe patients should play a shared role in the visit than attendings (mean 1.78 vs 1.83, *P*=.09). Results of the GLM showed no difference in scores by provider rank across time (*P*>.10).

## Discussion

### Principal Findings

This pilot study provided a unique opportunity to evaluate a brief 15-min simulated role-play conversation with a virtual patient or provider designed to promote effective communication and collaborative decision-making between health care providers and patients in order to improve intermediary health outcomes, including over-prescribing of antibiotics. Although there were not changes in activation scores for patients, the findings indicate that patients’ experienced short-term positive benefit on beliefs about antibiotic use and a positive, albeit intermediate, impact on patients’ knowledge about antibiotics. Patients with lower levels of activation, in particular, exhibited positive, short-term benefits in increased intent and confidence to discuss their needs and ask questions in the clinic visit and attitudes about engaging in shared decision-making with their provider. In particular, 79% of patients who saw their doctor after completing the simulation reported that it helped them in talking with their doctor. The results also suggest small immediate gains in providers’ attitudes about shared decision-making. Providers also felt that doctors would be better prepared and confident to have collaborative conversations with patients as well as create an environment conducive to active patient involvement in the encounter after completing the simulation. In particular, 77% of providers reported that the simulation had a positive impact on the way they now communicate with patients, 65% indicated that it helped them have a conversation with a patient about antibiotics, and 94% said they intent to further invite patients to ask questions and participate.

These findings support the role of utilizing simulated role-play conversations with virtual humans for the purpose of improving communication and relational (ie, empathy) skills in a variety of domains. Specifically, previous research has identified needed skill frameworks, training, practices, and elements of effective relationships that can be integrated in digital interventions to improve social emotional and communication skills, and drive positive behaviors [46]. Virtual simulations such as those used in this study, offer the ability to explore situations that would be stressful in person in a controlled environment to enhance the training and evaluation of critical communication skills [47]. Moreover, simulated role-play conversations with virtual humans allow an opportunity for extensive repetitive practice with feedback without consequence to a real patient, allowing for mastery learning [48]. Given these advantages, there has been an increase in the use of simulated role-play conversations with virtual humans to improve patient and provider communication in medical education as well as clinical settings. For example, the MYSELF project was developed to train providers in the expression and recognition of emotions and interpersonal communication skills through the use of an emotionally expressive virtual patient [49]. Other computer-based programs have been developed to improve pharmacy students’ motivational interviewing skills [50], medical students’ history-taking and basic communication skills [51], and promote healthy behaviors in patients with low health literacy [52].

A strength of this study was the use of an evidence-based simulation that leveraged virtual humans to improve users’ social emotional skills, empathy, motivational interviewing, and the use of sound communication tactics (ie, shared decision-making) that have been linked to sustained behavior change [53,54]. Moreover, the Kognito Conversations provide risk-free realistic role-plays that are sustainable and have high fidelity as opposed to face-to-face experiences, which are difficult to scale, expensive, and dependent on the skill and experience of each individual trainer and his or her knowledge of the population being trained. Finally, performing in front of others such as peers or instructors can increase the likelihood a trainee will feel embarrassment or social evaluative threat (ie, fear of being evaluated in a social setting) [55,56]. Both negative emotions in general and social evaluative threat in particular are known to impede cognitive performance [57-62]. Despite the many strengths of this approach, this pilot study offered several opportunities to improve on the simulation. For example, to mitigate the decline in knowledge experienced by patients at the 1-month follow-up visit, the final version of the simulation now includes a “teach-back” by the virtual doctor where learners are asked to explain in their own words why an antibiotic will not help a cold. The main points about antibiotic use are also now repeated within the simulation and in personalized feedback sessions through brief animated movies. To further improve communication skills, additional text was added to the performance dashboards, explaining the score the learner received in each area, and suggesting ways to do better in future visits. Changes were also made to draw learners’ attention to the Coach Advice button.

### Limitations

Several limitations of the study are worth noting. First, this was a single-group pre-post study. The lack of a control group limits our ability to attribute changes in participant’s behavioral intentions, attitudes, and perceptions of communication exclusively to the simulation. Moreover, it is possible that increased awareness from completing the presimulation assessments diminished our ability to detect significant changes in the postsimulation assessments. However, the primary focus of this pilot study was to establish the preliminary efficacy of this approach and not statistical significance. Relatedly, changes in scores from postsimulation to the 1-month follow-up may reflect a decay effect over time and not long-term change. The knowledge gained from this project will be used to develop the evidence for a larger randomized control trial. Second, the small sample size prohibits making any statistical inference generalizations about the study measures reliability (ie, alpha scores) and requires replication in a larger sample. Third, since the primary focus of this study was the use of a tablet-based simulation, a selection bias may be present whereby patients with low levels of computer literacy or poor vision may be underrepresented. To mitigate this risk, we implemented several strategies to increase the generalizability of this approach to all patient populations including the use of audio for the dialog and ensuring that the text was written at or below a 6th-grade reading level. Moreover, only 20% of individuals (13 patients and 4 providers) contacted to participate in the study declined, of which there were no differences in demographics between participants and nonparticipants; the most common reason for both patients and providers was lack of time. Fourth, participants were only permitted one opportunity to practice a one 10-15 min role-play conversation. Normally users have unlimited opportunities to practice multiple different conversations within a single simulation as well as opportunities to engage in these practice over time. Another important limitation is that the study design neither allows for definitive conclusions about whether the simulation affected patients’ actual level of engagement in their care nor whether shared decision-making as opposed to patient engagement was the primary communication strategy through which change occurred. Future studies should seek to disaggregate patient engagement from shared decision-making to elucidate the specific elements of communication that are associated with changes in patients’ knowledge and beliefs about antibiotic use. Moreover, future research should determine which elements of shared decision-making (ie, adequate information-exchange, taking into account patients’ values and preferences) are needed to improve patient outcomes. Preliminary results from this study suggest that patient-provider communication does not necessarily need to include patient participation in the final decision-making in order to be effective.

Finally, the external validity of our findings may be limited as a high percentage of the study participants (82%) were highly activated (as determined by PAM scores) at baseline (presimulation), even though the target audience for the simulation content was individuals with lower activation scores. This left little room for growth and could offer a plausible explanation for any nonsignificant findings. It is also plausible that the lack of significant findings was due to a baseline effect due to high levels of awareness about the problems with the overuse of antibiotics by patients and providers at the presimulation assessment.

In conclusion, this pilot study provided preliminary evidence on the efficacy of a simulation to improve patient-provider communication for engaging in collaborative conversations and decision-making on short-term improvements in patients’ knowledge and beliefs about antibiotic use. Future research should examine whether repeated opportunities for patients to use the simulation and practice the skills being taught may lead to sustained improvements in knowledge, beliefs, and behaviors. Moreover, although providers of all levels derived some benefits from the simulation, residents and medical students may experience the greatest gains in improving their communication skills for challenging conversations and attitudes about patient-centered care.

## Abbreviations

ABIM: America Board of Internal Medicine
API: application programming interface
CS-PAM: Clinician Support for Patient Activation Measure
GLM: Generalized Linear Models
MCCS: Medical Communication Competence Scale
PAM: Patient Activation Measure
PPOS: Provider Orientation Scale
RWJF: Robert Wood Johnson Foundation
SD: standard deviation
SDM: shared decision-making

## References

[1] Mainous AG, Diaz VA, Matheson EM, Gregorie SH, Hueston WJ (2011). Trends in hospitalizations with antibiotic-resistant infections: U.S., 1997-2006. Public Health Rep.

[2] Dowell J, Hudson H (1997). A qualitative study of medication-taking behaviour in primary care. Fam Pract.

[3] Blaser M (2011). Antibiotic overuse: stop the killing of beneficial bacteria. Nature.

[4] Trasande L, Blustein J, Liu M, Corwin E, Cox L, Blaser M (2013). Infant antibiotic exposures and early-life body mass. Int J Obes (Lond).

[5] Pijpers E, Kuyvenhoven M, Tonkin-Crine S, Little P, Verheij T, van der Velden AW (2012). Effectiveness of physician-targeted interventions to improve antibiotic use for respiratory tract infections. Br J Gen Pract.

[6] Van Boeckel TP, Gandra S, Ashok A, Caudron Q, Grenfell BT, Levin SA, Laxminarayan R (2014). Global antibiotic consumption 2000 to 2010: an analysis of national pharmaceutical sales data. Lancet Infect Dis.

[7] Fleming-Dutra KE, Hersh AL, Shapiro DJ, Bartoces M, Enns EA, File TM, Finkelstein JA, Gerber JS, Hyun DY, Linder JA, Lynfield R, Margolis DJ, May LS, Merenstein D, Metlay JP, Newland JG, Piccirillo JF, Roberts RM, Sanchez GV, Suda KJ, Thomas A, Woo TM, Zetts RM, Hicks LA (2016). Prevalence of inappropriate antibiotic prescriptions among US ambulatory care visits, 2010-2011. JAMA.

[8] Dempsey P, Businger A, Whaley L, Gagne J, Linder J (2014). Primary care clinicians' perceptions about antibiotic prescribing for acute bronchitis: a qualitative study. BMC Fam Pract.

[9] Tonkin-Crine S, Yardley L, Little P (2011). Antibiotic prescribing for acute respiratory tract infections in primary care: a systematic review and meta-ethnography. J Antimicrob Chemother.

[10] Gonzales R, Steiner JF, Lum A, Barrett PH (1999). Decreasing antibiotic use in ambulatory practice: impact of a multidimensional intervention on the treatment of uncomplicated acute bronchitis in adults. JAMA.

[11] Flottorp S, Oxman AD, Håvelsrud K, Treweek S, Herrin J (2002). Cluster randomised controlled trial of tailored interventions to improve the management of urinary tract infections in women and sore throat. BMJ.

[12] Coxeter P, Del Mar Chris B, McGregor L, Beller EM, Hoffmann TC (2015). Interventions to facilitate shared decision making to address antibiotic use for acute respiratory infections in primary care. Cochrane Database Syst Rev.

[13] (2016). Choosing Wisely.

[14] (2013). Health Affairs.

[15] Greene J, Hibbard JH (2012). Why does patient activation matter? an examination of the relationships between patient activation and health-related outcomes. J Gen Intern Med.

[16] Jordan M, Lanham H, Crabtree B (2009). The role of conversation in health care interventionsnabling sensemaking and learning. Implement Sci.

[17] Coiera E (2000). When conversation is better than computation. J Am Med Inform Assoc.

[18] Albright GL, Davidson J, Goldman R, Shockley KM, Timmons-Mitchell J (2016). Development and validation of the gatekeeper behavior scale. Crisis.

[19] Lane C, Rollnick S (2007). The use of simulated patients and role-play in communication skills training: a review of the literature to August 2005. Patient Educ Couns.

[20] Rosenbaum M, Ferguson K, Lobas J (2004). Teaching medical students and residents skills for delivering bad news: a review of strategies. Acad Med.

[21] Andrade AD, Bagri A, Zaw K, Roos BA, Ruiz JG (2010). Avatar-mediated training in the delivery of bad news in a virtual world. J Palliat Med.

[22] Aspegren K (1999). BEME Guide No. 2: teaching and learning communication skills in medicine-a review with quality grading of articles. Med Teach.

[23] Joyner B, Young L (2006). Teaching medical students using role play: twelve tips for successful role plays. Med Teach.

[24] Miller WR (2012). Motivational Interviewing: Helping People Change, 3rd edition.

[25] Bateman A, Fonagy P (2010). Mentalization based treatment for borderline personality disorder. World Psychiatry.

[26] Bateman AW, Fonagy P (2004). Mentalization-based treatment of BPD. J Pers Disord.

[27] Allen JG, Fonagy P (2006). The Handbook of Mentalization-Based Treatment.

[28] Decety J, Jackson PL (2004). The functional architecture of human empathy. Behav Cogn Neurosci Rev.

[29] Stotland E (1966). Mentalism revisited. J Gen Psychol.

[30] Ickes W (1993). Empathic accuracy. J Personal.

[31] Ochsner K, Silvers J, Buhle J (2012). Functional imaging studies of emotion regulation: a synthetic review and evolving model of the cognitive control of emotion. Ann N Y Acad Sci.

[32] Gross J (1998). Rev Gen Psychol.

[33] Bandura A (2001). Social cognitive theory of mass communication. Media Psychol.

[34] Lim KK, Teh CC (2012). A cross sectional study of public knowledge and attitude towards antibiotics in Putrajaya, Malaysia. South Med Rev.

[35] Hibbard JH, Mahoney ER, Stockard J, Tusler M (2005). Development and testing of a short form of the patient activation measure. Health Serv Res.

[36] Hibbard JH, Stockard J, Mahoney ER, Tusler M (2004). Development of the patient activation measure (PAM): conceptualizing and measuring activation in patients and consumers. Health Serv Res.

[37] Hibbard JH, Collins PA, Mahoney E, Baker LH (2010). The development and testing of a measure assessing clinician beliefs about patient self-management. Health Expect.

[38] Krupat E, Hsu J, Irish J, Schmittdiel JA, Selby J (2004). Matching patients and practitioners based on beliefs about care: results of a randomized controlled trial. Am J Manag Care.

[39] Shaw WS, Woiszwillo MJ, Krupat E (2012). Further validation of the patient-practitioner orientation scale (PPOS) from recorded visits for back pain. Patient Educ Couns.

[40] Scholl I, Kriston L, Dirmaier J, Buchholz A, Härter M (2012). Development and psychometric properties of the shared decision making questionnaire--physician version (SDM-Q-Doc). Patient Educ Couns.

[41] Simon D, Schorr G, Wirtz M, Vodermaier A, Caspari C, Neuner B, Spies C, Krones T, Keller H, Edwards A, Loh A, Härter M (2006). Development and first validation of the shared decision-making questionnaire (SDM-Q). Patient Educ Couns.

[42] Cegala DJ, Coleman MT, Turner JW (1998). The development and partial assessment of the medical communication competence scale. Health Commun.

[43] Fishbein M, Ajzen I (1975). Belief, Attitude, Intention, and Behavior: An Introduction to Theory and Research.

[44] Bandura A (1977). Self-efficacy: toward a unifying theory of behavioral change. Psychol Rev.

[45] Arozullah AM, Yarnold PR, Bennett CL, Soltysik RC, Wolf MS, Ferreira RM, Lee SD, Costello S, Shakir A, Denwood C, Bryant FB, Davis T (2007). Development and validation of a short-form, rapid estimate of adult literacy in medicine. Med Care.

[46] Lateef F (2010). Simulation-based learning: just like the real thing. J Emerg Trauma Shock.

[47] Mantovani F, Castelnuovo G, Gaggioli A, Riva G (2003). Virtual reality training for health-care professionals. Cyberpsychol Behav.

[48] McGaghie WC, Siddall VJ, Mazmanian PE, Myers J, American College of Chest Physicians HealthScience Policy Committee (2009). Lessons for continuing medical education from simulation research in undergraduate and graduate medical education: effectiveness of continuing medical education: American College of Chest Physicians Evidence-Based Educational Guidelines. Chest.

[49] Anolli L, Mantovani F, Balestra M, Agliati A, Realdon O, Zurloni V, Mortillaro M, Vescovo A, Confalonieri L (2004). Researchgae.

[50] Villaume WA, Berger BA, Barker BN (2006). Learning motivational interviewing: scripting a virtual patient. Am J Pharm Educ.

[51] Stevens A, Hernandez J, Johnsen K, Dickerson R, Raij A, Harrison C, DiPietro M, Allen B, Ferdig R, Foti S, Jackson J, Shin M, Cendan J, Watson R, Duerson M, Lok B, Cohen M, Wagner P, Lind DS (2006). The use of virtual patients to teach medical students history taking and communication skills. Am J Surg.

[52] Bickmore TW, Silliman RA, Nelson K, Cheng DM, Winter M, Henault L, Paasche-Orlow MK (2013). A randomized controlled trial of an automated exercise coach for older adults. J Am Geriatr Soc.

[53] Albright G, Adam C (2016). Kognito.

[54] Albright G, Adam C, Goldman R, Serri D (2013). A game-based simulation utilizing virtual humans to train physicians to screen and manage the care of patients with mental health disorders. Games Health J.

[55] Nestel D, Tierney T (2007). Role-play for medical students learning about communication: guidelines for maximising benefits. BMC Med Educ.

[56] Stevenson K, Sander P (2002). Medical students are from Mars--business and psychology students are from Venus--- University teachers are from Pluto?. Med Teach.

[57] Baumeister R, Twenge J, Nuss C (2002). Effects of social exclusion on cognitive processes: anticipated aloneness reduces intelligent thought. J Pers Soc Psychol.

[58] Bolte A, Goschke T, Kuhl J (2003). Emotion and intuition. Psychol Sci.

[59] Kuhlmann S, Piel M, Wolf OT (2005). Impaired memory retrieval after psychosocial stress in healthy young men. J Neurosci.

[60] Lupien SJ, Gaudreau S, Tchiteya BM, Maheu F, Sharma S, Nair NP, Hauger RL, McEwen BS, Meaney MJ (1997). Stress-induced declarative memory impairment in healthy elderly subjects: relationship to cortisol reactivity. J Clin Endocrinol Metab.

[61] Payne JD, Jackson ED, Ryan L, Hoscheidt S, Jacobs JW, Nadel L (2006). The impact of stress on neutral and emotional aspects of episodic memory. Memory.

[62] Smallwood J, Fitzgerald A, Miles LK, Phillips LH (2009). Shifting moods, wandering minds: negative moods lead the mind to wander. Emotion.

